# Supplementary material for: Trpv4 links environmental temperature to testicular differentiation in hermaphroditic ricefield eel
Source: eLife. 2026 Sep 7;14:RP108272. doi: 10.7554/eLife.108272 (PMC13549621; doi:10.7554/eLife.108272)
Supplement: Supplementary file 9 [file elife-108272-fig3-figsupp1-data1.zip › Figure 3-figure supplement 1-source data 1/Figure 3-figure supplement 1-source data 1.pdf]

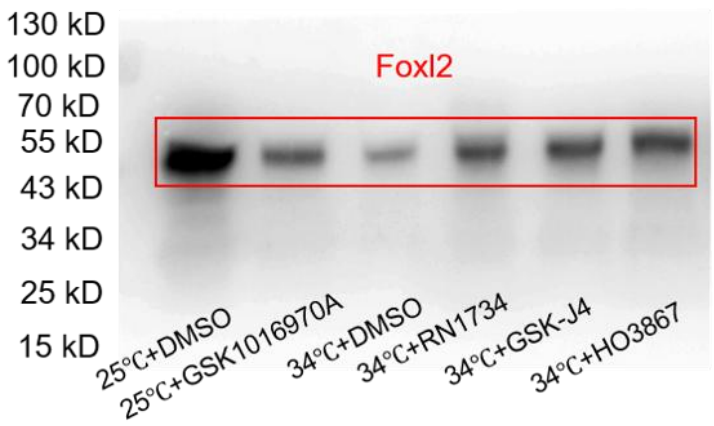
130 kD
100 kD
70 kD
55 kD
43 kD
34 kD
25 kD
15 kD
Foxl2
25℃+DMSO
25℃+GSK1016970A
34℃+DMSO
34℃+RN1734
34℃+GSK-J4
34℃+HO3867

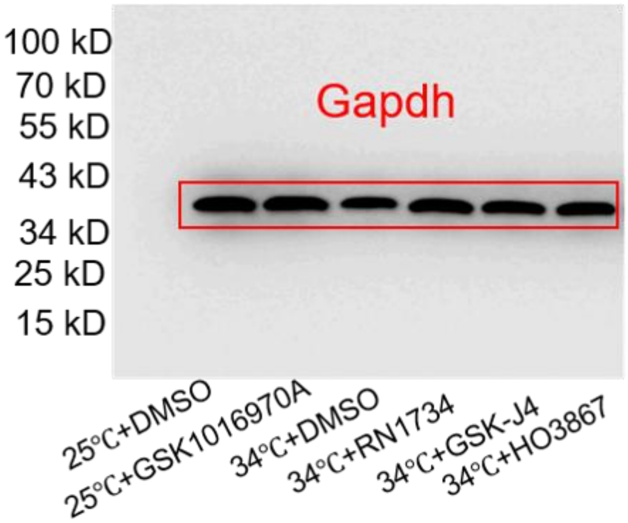
100 kD
70 kD
55 kD
43 kD
34 kD
25 kD
15 kD
Gapdh
25℃+DMSO
25℃+GSK1016970A
34℃+DMSO
34℃+RN1734
34℃+GSK-J4
34℃+HO3867

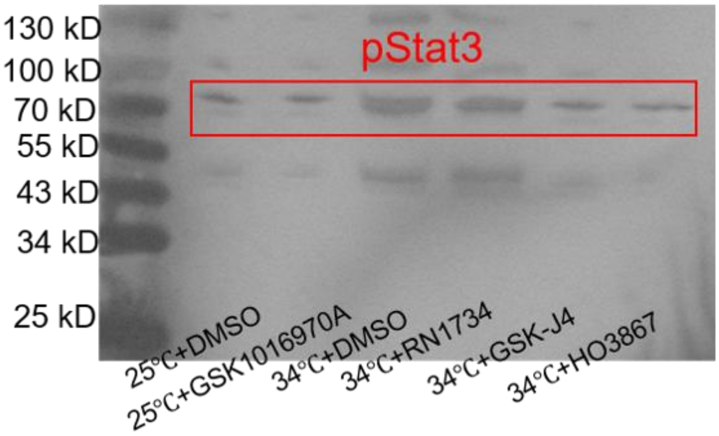
130 kD
100 kD
70 kD
55 kD
43 kD
34 kD
25 kD
pStat3
25℃+DMSO
25℃+GSK1016970A
34℃+DMSO
34℃+RN1734
34℃+GSK-J4
34℃+HO3867

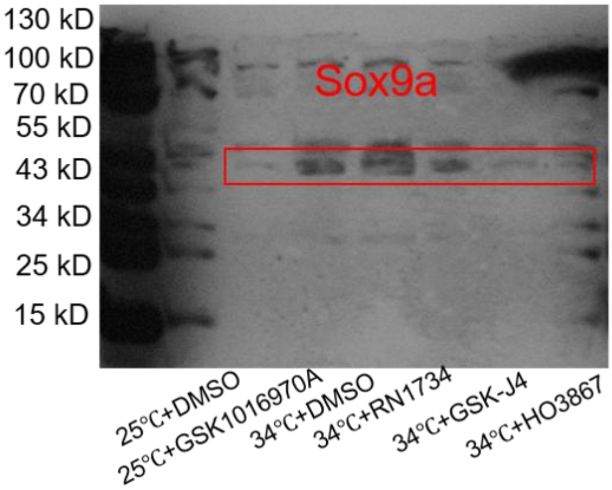

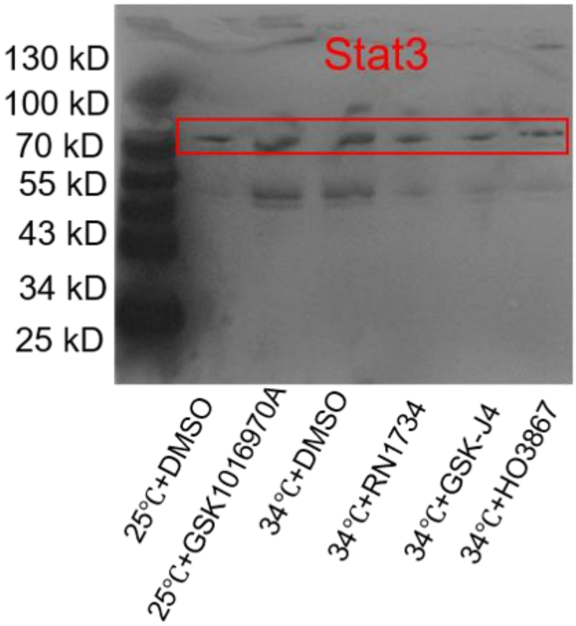

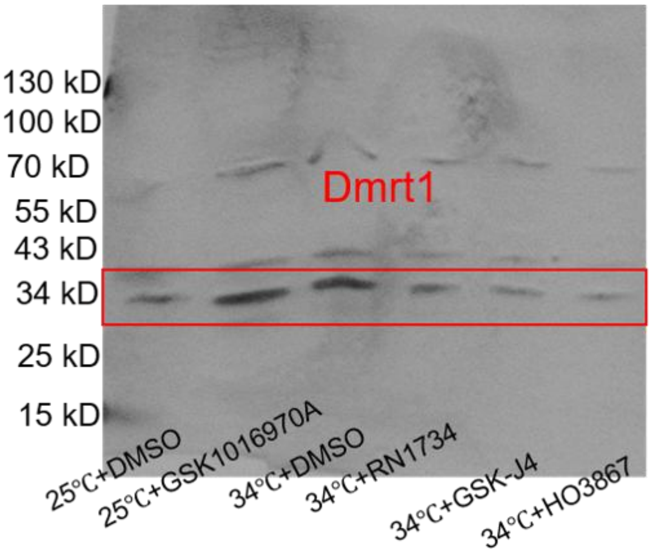

Figure 3-figure supplement 1-source data 1. PDF file containing original western blots for Figure 3-figure supplement 1C, indicating the relevant bands and treatments.
