# Supplementary material for: Trpv4 links environmental temperature to testicular differentiation in hermaphroditic ricefield eel
Source: eLife. 2026 Sep 7;14:RP108272. doi: 10.7554/eLife.108272 (PMC13549621; doi:10.7554/eLife.108272)
Supplement: Supplementary file 12 [file elife-108272-fig3-figsupp2-data1.zip › Figure 3-figure supplement 2-source data 1/Figure 3-figure supplement 2-source data 1.pdf]

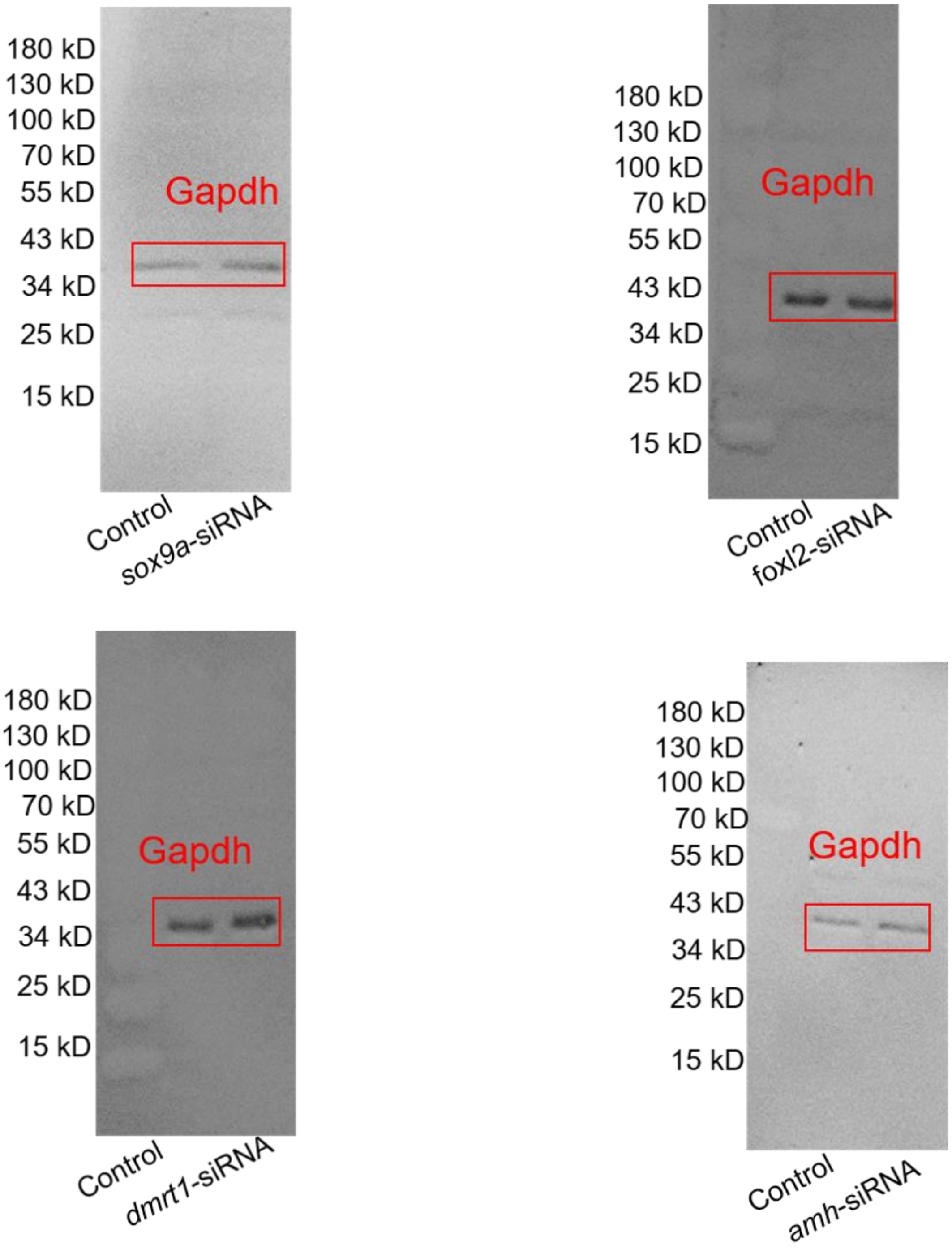
180 kD
130 kD
100 kD
70 kD
55 kD
43 kD
34 kD
25 kD
15 kD
Gapdh
Control
sox9a-siRNA
Gapdh
Control
foxl2-siRNA
Gapdh
Control
dmrt1-siRNA
Gapdh
Control
amh-siRNA

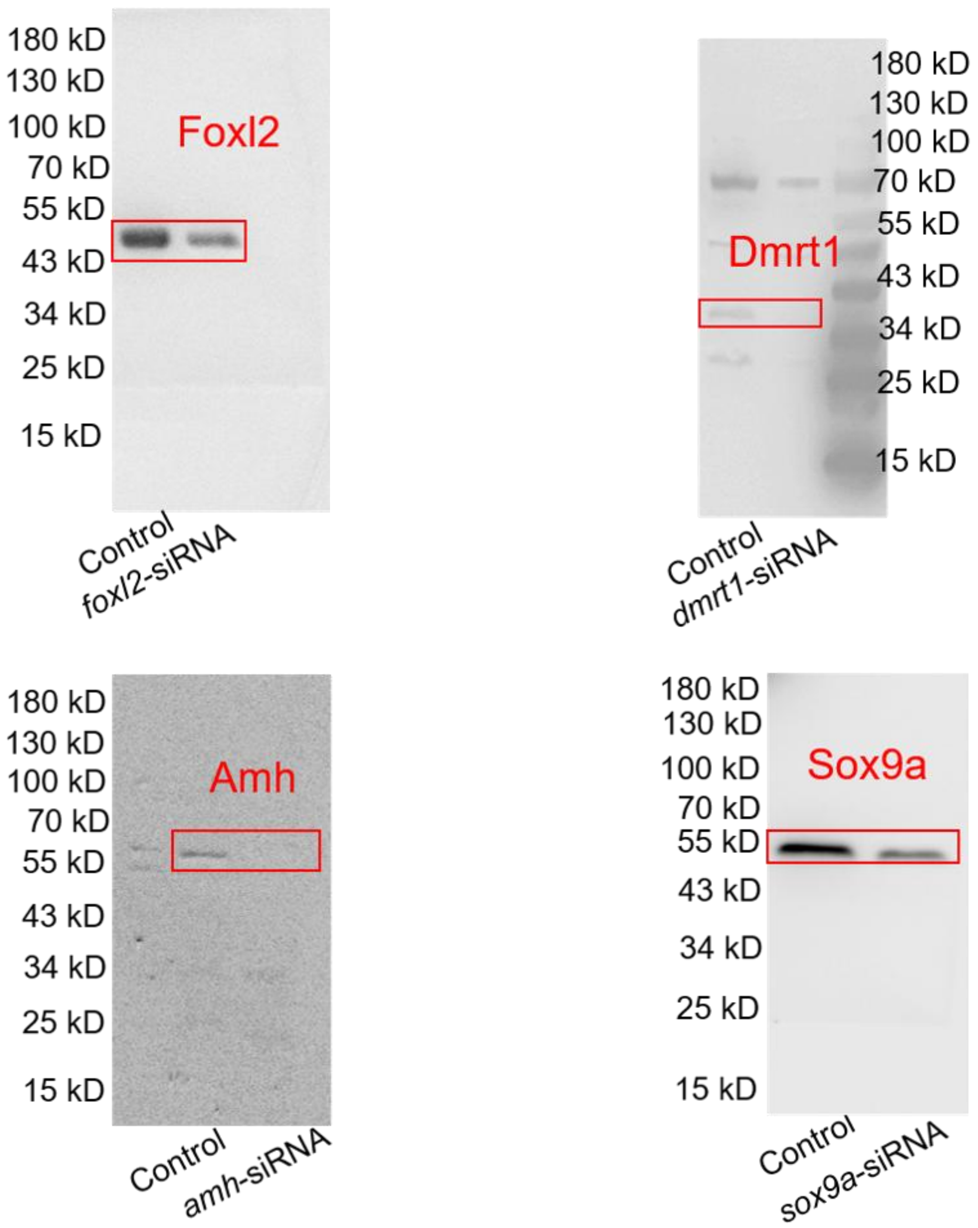

Figure 3-figure supplement 2-source data 1. PDF file containing original western blots for Figure 3-figure supplement 2A, indicating the relevant bands and treatments.
