# Supplementary material for: Trpv4 links environmental temperature to testicular differentiation in hermaphroditic ricefield eel
Source: eLife. 2026 Sep 7;14:RP108272. doi: 10.7554/eLife.108272 (PMC13549621; doi:10.7554/eLife.108272)
Supplement: Supplementary file 15 [file elife-108272-fig4-data1.zip › Figure 4-source data 1/Figure 4-source data 1.pdf]

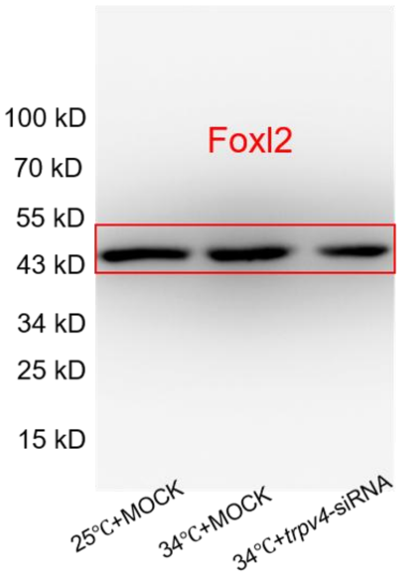
100 kD
70 kD
55 kD
43 kD
34 kD
25 kD
15 kD
Foxl2
25°C+MOCK
34°C+MOCK
34°C+trpv4-siRNA

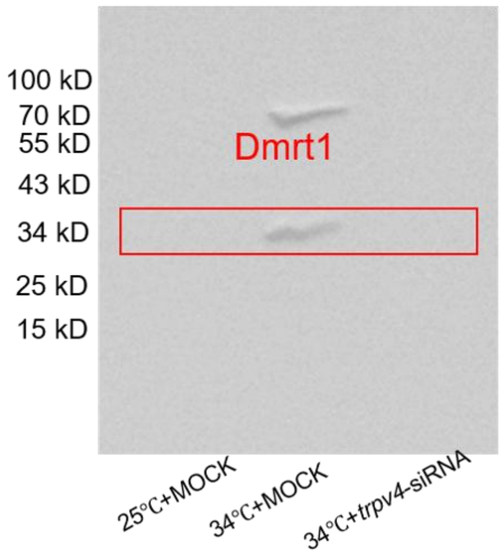
100 kD
70 kD
55 kD
43 kD
34 kD
25 kD
15 kD
Dmrt1
25°C+MOCK
34°C+MOCK
34°C+trpv4-siRNA

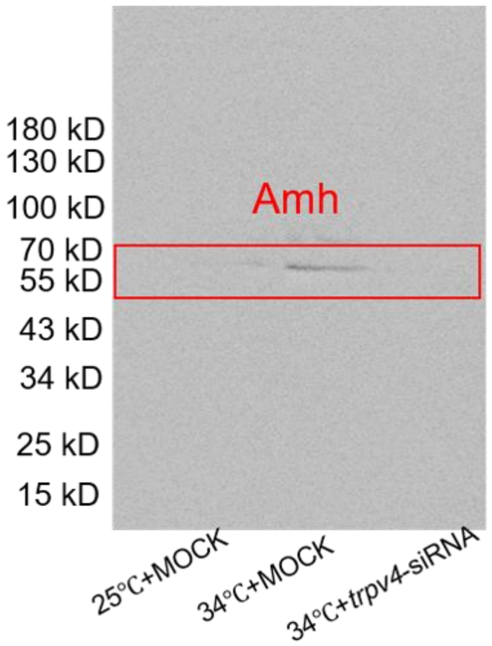
180 kD
130 kD
100 kD
70 kD
55 kD
43 kD
34 kD
25 kD
15 kD
Amh
25°C+MOCK
34°C+MOCK
34°C+trpv4-siRNA

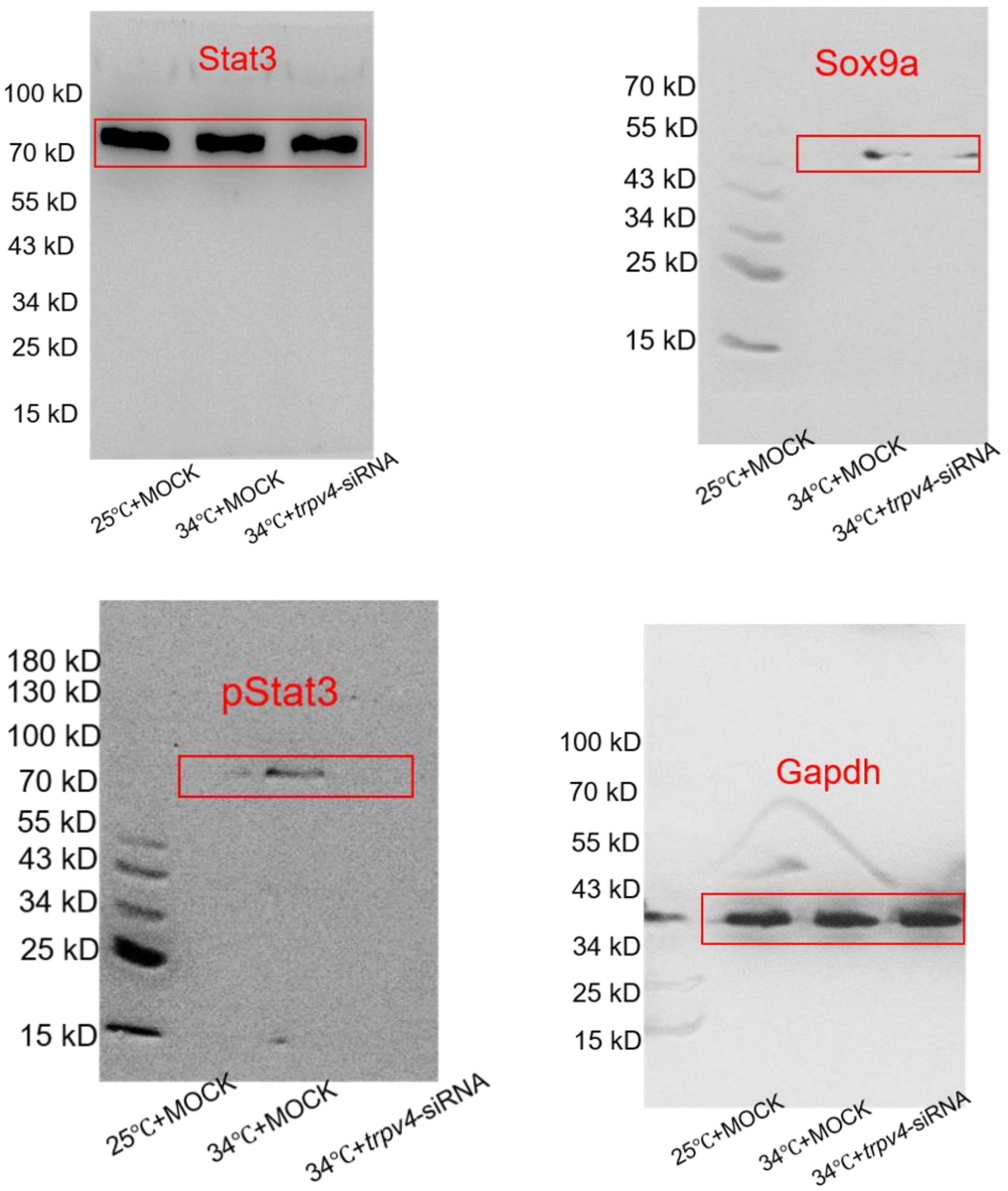

Figure 4-source data 1. PDF file containing original western blots for Figure 4B, indicating the relevant bands and treatments.
