# Supplementary material for: Trpv4 links environmental temperature to testicular differentiation in hermaphroditic ricefield eel
Source: eLife. 2026 Sep 7;14:RP108272. doi: 10.7554/eLife.108272 (PMC13549621; doi:10.7554/eLife.108272)
Supplement: Supplementary file 18 [file elife-108272-fig5-data1.zip › Figure 5-source data 1/Figure 5-source data 1.pdf]

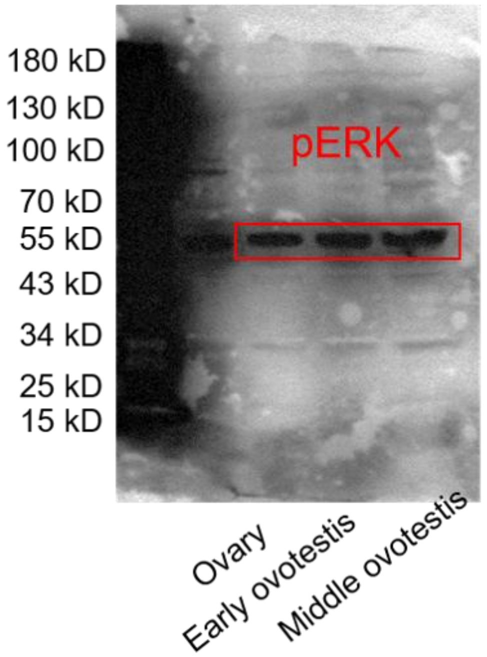
180 kD
130 kD
100 kD
70 kD
55 kD
43 kD
34 kD
25 kD
15 kD
pERK
Ovary
Early ovotestis
Middle ovotestis

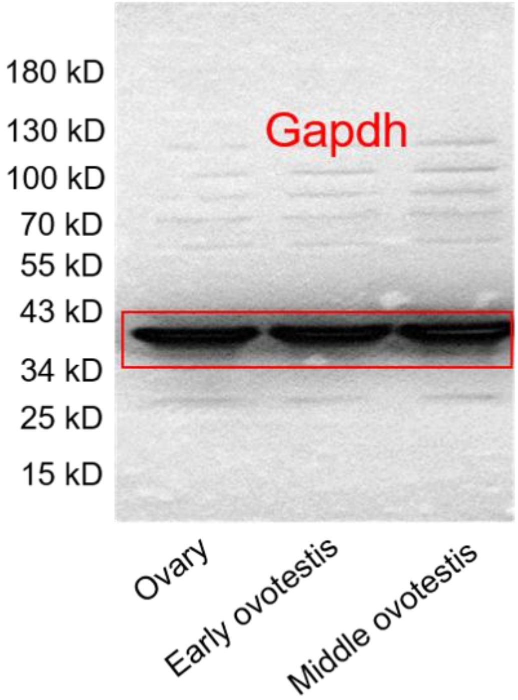
180 kD
130 kD
100 kD
70 kD
55 kD
43 kD
34 kD
25 kD
15 kD
Gapdh
Ovary
Early ovotestis
Middle ovotestis

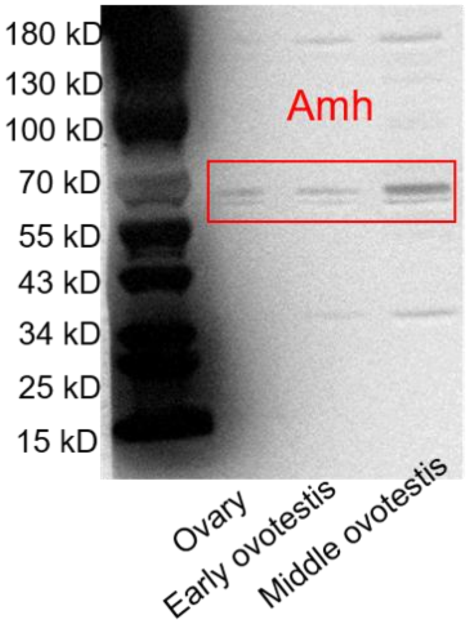
180 kD
130 kD
100 kD
70 kD
55 kD
43 kD
34 kD
25 kD
15 kD
Amh
Ovary
Early ovotestis
Middle ovotestis

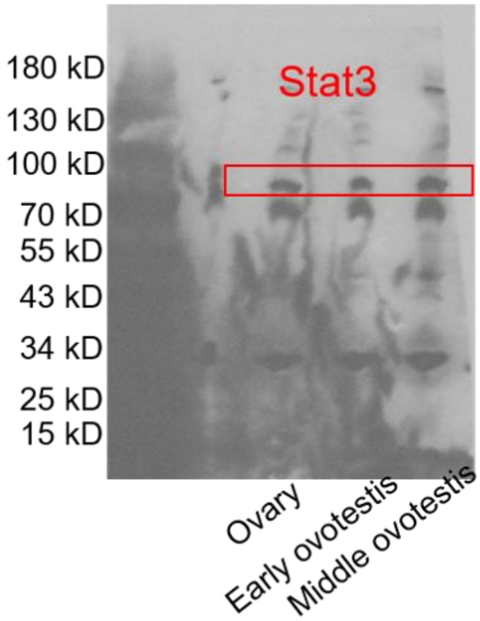

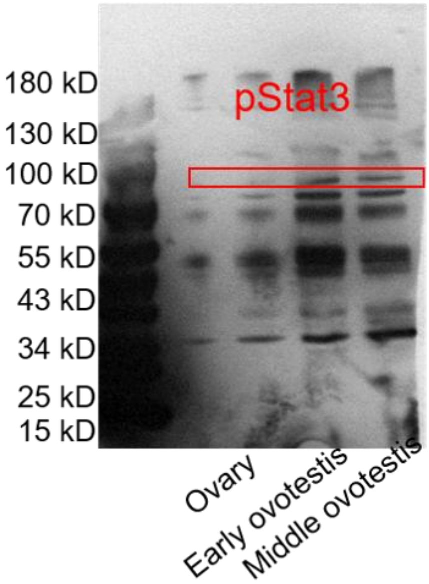

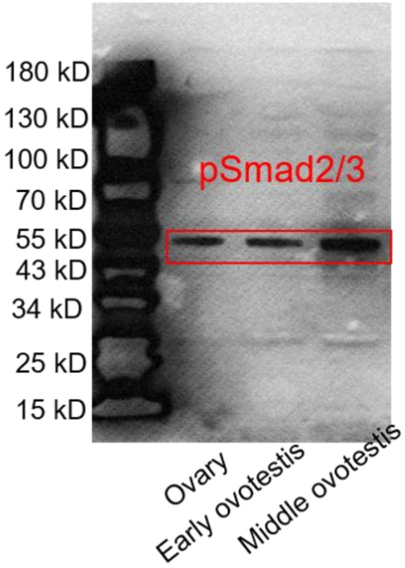

Figure 5-source data 1. PDF file containing original western blots for Figure 5E, indicating the relevant bands and treatments.
