# Supplementary material for: Trpv4 links environmental temperature to testicular differentiation in hermaphroditic ricefield eel
Source: eLife. 2026 Sep 7;14:RP108272. doi: 10.7554/eLife.108272 (PMC13549621; doi:10.7554/eLife.108272)
Supplement: Supplementary file 20 [file elife-108272-fig5-data3.zip › Figure 5-source data 3/Figure 5-source data 3.pdf]

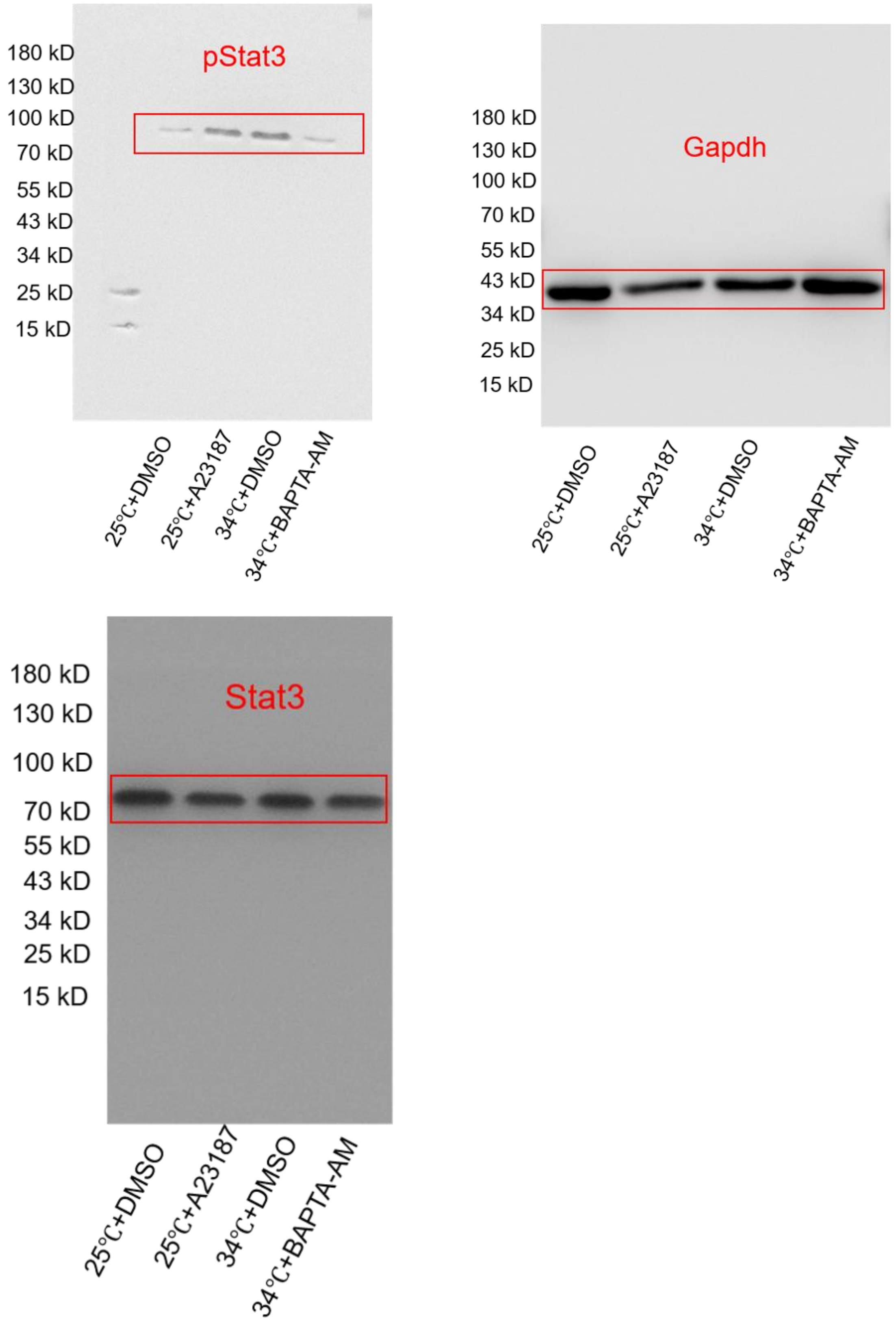

Figure 5-source data 3. PDF file containing original western blots for Figure 5G, indicating the relevant bands and treatments.
