# Supplementary material for: Trpv4 links environmental temperature to testicular differentiation in hermaphroditic ricefield eel
Source: eLife. 2026 Sep 7;14:RP108272. doi: 10.7554/eLife.108272 (PMC13549621; doi:10.7554/eLife.108272)
Supplement: Peer review history

# Editor report: eLife Assessment

**DOI:** 10.7554/eLife.108272.4.sa0

**Authors:** Erika A Bach

**Affiliations:** NYU Grossman School of Medicine United States

**Keywords:** Incomplete, Useful

This study presents **useful** findings on the molecular mechanisms driving female-to-male sex reversal in the ricefield eel (Monopterus albus) during aging, which would be of interest to biologists studying sex determination. The manuscript describes an interesting mechanism potentially underlying sex differentiation in M. albus. However, the current data are **incomplete** and would benefit from more rigorous experimental approaches for Western blotting.

# Referee report: Reviewer #1 (Public review)

**DOI:** 10.7554/eLife.108272.4.sa1

Summary:

This preprint investigates the molecular mechanism by which warm temperature induces female-to-male sex reversal in the ricefield eel (Monopterus albus), a protogynous hermaphroditic fish of significant aquacultural value in China. The study identifies Trpv4 - a temperature-sensitive Ca²⁺ channel - as a putative thermosensor linking environmental temperature to sex determination. The authors propose that Trpv4 causes Ca²⁺ influx, leading to activation of Stat3 (pStat3). pStat3 then transcriptionally upregulates the histone demethylase Kdm6b (aka Jmjd3), leading to increased dmrt1 gene expression and ovo-testes development. This work aims to bridge ecological cues with molecular and epigenetic regulators of sex change and has potential implications for sex control in aquaculture.

This revision is an improvement to the manuscript. However, there are still several remaining issues that are not resolved and that limit enthusiasm.

(1) The Supplementary File 1 contains a compilation of Western blots. However, the control protein (for example GAPDH) is on a *different gel* in all of the tabs. For best practices, the protein that is used as the "loading control" needs to be on the same membrane (same Western blot), not on a different blot. It is not compelling to normalize a loading control protein on a separate blot. This reduces enthusiasm for all of the protein data in the manuscript.

a. The blots under the tab "Fig. 5D" are dirty and the blot the GAPDH is over-exposed.

(2) The images provided in the response to authors have no legends and are not explained in the text. As such, they are not supportive data in their current form.

(3) The antibodies that were listed as "home-made" need to be described in great details. For example, we need to know the species that the antibodies were generated in. Additionally, we need to know the antigen (amino acid residues of the recombinant protein).

(4) The reference genes for the qRT-PCR are not listed in the Materials and Methods. The authors need to list the reference gene and tell us why they selected those genes.

(5) The comparison of the turtle and ricefield eel of kdm6b should be shown as a supplementary file and not listed as data not shown.

# Author comment: Author response

**DOI:** 10.7554/eLife.108272.4.sa2

**Authors:** Yuhua Sun, Zhi Yang, Yimin Zhang, Tingting Luo

**Affiliations:** Institute of Hydrobiology WUhan China; Institute of Hydrobiology WUhan China; Institute of Hydrobiology WUhan China; Institute of Hydrobiology WUhan China

The following is the authors’ response to the previous reviews

**Reviewer #1 (Public review):**

Summary:

This preprint investigates the molecular mechanism by which warm temperature induces female-to-male sex reversal in the ricefield eel (Monopterus albus), a protogynous hermaphroditic fish of significant aquacultural value in China. The study identifies Trpv4 - a temperature-sensitive Ca²⁺ channel - as a putative thermosensor linking environmental temperature to sex determination. The authors propose that Trpv4 causes Ca²⁺influx, leading to activation of Stat3 (pStat3). pStat3 then transcriptionally upregulates the histone demethylase Kdm6b (aka Jmjd3), leading to increased dmrt1 gene expression and ovo-testes development. This work aims to bridge ecological cues with molecular and epigenetic regulators of sex change and has potential implications for sex control in aquaculture.

Strengths:

(1) This study proposes the first mechanistic pathway linking thermal cues to natural sex reversal in adult ricefield eel, extending the temperature-dependent sex determination paradigm beyond embryonic reptiles and saltwater fish

(2) The findings could have applications for aquaculture, where skewed sex ratios apparently limit breeding efficiency

Weaknesses:

Although the revised manuscript represents an improvement over the original version, substantial weaknesses remain.

We thank you for the critical comments. We have responded to your concerns by a point by point manner, and please see detail below.

Scientific Concerns

(1) Western blot normalization and exposure: The loading controls (GAPDH) in Fig. S3C appear overexposed, as do several Foxl2 blots. Because these signals are likely outside the linear range, I am not convinced that normalization is reliable. This raises concerns about the validity of the quantified results.

We thank you for the concerns. We have repeated the experiments, and new blots were loaded in Fig.S3C.

(2) Antibody validation and referencing (Line 776): The authors need to refer explicitly to figures demonstrating antibody validation. At present, these data are provided only as a supplementary file that is not cited in the manuscript. In addition, the Sox9a antibody appears to yield indistinguishable signals in control and RNAi conditions, suggesting that it may not recognize eel Sox9a. This issue is not addressed by the authors. Furthermore, antibody validation Western blots should be quantified.

We thank you for the comments. We have repeated the siRNA experiments to show the specificity of the antibodies used. This file, named as the supplementary file 1, is now cited in “WB analysis” in the Materials and Method part. As required, the antibody validation of WB are uploaded in the supplementary file 1. Antibody validation for WB are now quantified, and please see the new figure 3 and supplementary Figure 3.

(3) Unclear sample sizes (N values): Sample sizes remain unclear for several figures:

(a) Fig. 3F - No N value is provided. Each graph shows three data points; does this indicate that only three samples were quantified? If ten samples were collected, why were all not quantified?

We apologize for the confusion. Three data points were previously used to shown data of 3 replicates. In new figure 3F, 10 randomly selected sections were imaged, and the data are shown. In the revised manuscript, the sample numbers (the N values) are added, and all the information can be found in the figure legend.

(b) Fig. 4 - No N values are reported.

Now N values are added. Please see the figure legend.

(c) Fig. 5A - Again, only three data points are shown per group, despite the apparent availability of twelve samples. The rationale for this discrepancy is not explained.

We apologize for the wrong data representation. Now all the data points are shown in Figure 5.

(4) qRT-PCR normalization: The manuscript does not specify the reference gene(s) used for qRT-PCR normalization. Although expression levels are reported as "relative," neither the identity of the reference gene(s) nor the justification for their selection is provided.

We now have specify the reference gene in “Quantitative real-time PCR (qPCR) experiments” part in the Materials and Methods section.

(5) Specificity of key antibodies: While the authors have made some effort to validate anti-Amh, anti-Sox9, and anti-Dmrt antibodies, the results remain incomplete. The Amh and Dmrt antibodies detect reduced protein levels following knockdown of their respective targets, which is encouraging. However, the Sox9a antibody shows no difference between control and RNAi conditions, suggesting it does not recognize eel Sox9. This is not acknowledged in the manuscript. In addition, no validation data are presented for Foxl2. Antibody validation data must be clearly referenced in the main text and presented in an interpretable and quantitative manner.

The antibody specificity is very important. For that reason, we have generated at least two different antibodies for each target protein, using full-length or small peptide as antigen. We have repeated the experiments for key antibodies such as Dmrt1 and Sox9a. IF and WB results clearly showed the specificity of the antibodies.

**Author response image 1.** 

Foxl2 antibody has also been reported in ricefield eel (Hu et al. SCIENTIFIC REPORTS | 4: 6884 | DOI: 10.1038/srep06884, Molecular cloning and analysis of gonadal expression of Foxl2 in the ricefield eel *Monopterus albus*).

After short term warm temperature exposure, only a small portion of somatic cells in ovary may be induced to express the male markers. As different techniques have different capacity (sensitivity), some techniques were more easy to detect that change. For instance, qPCR and WB are ready to detect it, whereas IF is a little difficult in obtaining good quality data.

(6) Immunofluorescence data quality: The immunofluorescence images remain difficult to interpret. I strongly encourage the authors to enlarge the image panels and to present monochrome images (white signal on black background). The current presentation severely limits interpretability.

We thank you for the comments. We think that our IF images are of decent quality. Due to the limits of the Figure space (already busy for Figure 3), enlarging the image panels or presenting additional monochrome images will compromise the quality of other data. Alternatively, if you still concern its quality, we can put it in the supplementary.

**Author response image 2.**

(7) Unreferenced supplementary figure: Fig. S4 is included in the submission but is not referenced anywhere in the manuscript text.

We now have renamed the supplementary Figures. And we have double checked the text to make sure all Figure information is correctly referenced. Figure S4 is removed, as it is not necessary.

(8) Fig. 5B image resolution: The micrographs in Fig. 5B are too small to allow meaningful evaluation of the data.

Now new Figure 5B images with higher resolution were shown.

(9) Unexplained data inclusion (Fig. 5E): Fig. 5E includes a pERK blot that is not mentioned in the Results section. The rationale for including these data is unclear.

Previous work have shown that FGF/ERK signaling may play a role in sex change of ricefield eel (in Chinese). We therefore examined the Erk activity to explore whether it is involved in sex reversal. The results showed that pErk was comparable between ovary and ovotestis. At your suggestion, we decided to remove the data.

(10) Poor blot quality (Fig. S3C): The blots in Fig. S3C exhibit high background and overexposure. I am concerned about the reliability of the quantification shown in panel D.

The experiments have been repeated at least three times, and similar results were obtained. We now have replaced some of the WB that were of high background or overexposure.

(11) Poor blot quality (Fig. S5G): The Stat3 blots in Fig. S5G contain numerous white artifacts, raising concerns about their suitability for normalization in panel H.

We now have repeated the experiments, and uploaded a new representative blot with better quality.

(12) Missing controls (Fig. 6E): Fig. 6E lacks controls for HO-3867 and Colivelin treatments alone. Without these controls, it is not possible to determine whether the reported effects are meaningful.

We thank you for the comments. We now have added the data required (with HO-3867 and Colivelin treatments alone).

(13) Graphical presentation: The use of a light blue-to-pink gradient in bar graphs throughout the manuscript does not aid interpretation. I recommend using more distinct colors (e.g., red, orange, green, blue, purple, gray, black) to improve clarity.

We thank you for the comments. We now have changed the blue-to-pink gradient to more distinct color system to better present the data. Please see the detail in the revised Figures.

In summary, the interpretation of the study remains limited by persistent issues related to data presentation, image quality, and reagent specificity.

We thank you for the critical comments about our data, in particular for antibody specificity and image quality, and the detailed instruction for how to better present the data. Answering your questions have greatly improved the quality of the manuscript. We admit that due to the technique challenging (with different conditions and different doses of small molecules) and higher cost of animal experiments, some of the WB or IF experiments may not be of high standards.

**Recommendations for the authors:**

**Reviewer #1 (Recommendations for the authors):**

**Editorial Concerns**

(1) Overstatement of conclusions: In lines 16-18, the authors state that Trpv4 "mediates" warm temperature-driven sex reversal. This claim is too strong given the data and should be toned down.

We agree with our editorial comment about the overstatement. Now it reads “Trpv4 links environmental temperature to testicular differentiation in ricefield eel”.

(2) Misuse of statistical language (Line 213): The term "significant" is used where statistical significance was not measured. The wording should be revised.

We thank you for the point, and now have replaced “significant” to “marked”.

(3) Terminology (Line 238): The term "co-expression" is inaccurate in this context. I suggest replacing it with "co-upregulation."

We thank you for the point, and have changed it accordingly.

(4) Drug description errors (Lines 241-242): The manuscript incorrectly identifies which drug functions as an agonist and which as an antagonist. This caused considerable confusion and must be corrected.

We have carefully checked the sentence, and it was correct, as RN1734 and GSK1016790A are known Trpv4 specific antagonist and agonist, respectively.

(5) Gene examples missing (Lines 247-250): The authors should explicitly name the testis-biased and ovary-biased genes referred to in this section.

We thank you for the point, and now it reads “warm temperature exposure increased the expression of testicular differentiation genes such as dmrt1 and gsdf, accompanied by moderately decreased expression of ovarian differentiation genes such as cyp19a1a and foxl2”.

(6) Lack of experimental context (Lines 322-324): Rather than simply listing the drugs used, the authors should briefly explain what each compound inhibits or activates and why it was employed.

We have described this in the manuscript. The information of pStat3 activator and inhibitor has been described in Lines 305-309, as “HO-3867, a curcumin analogue, is a selective pStat3 inhibitor, which blocks pStat3 activity by directly binding to Stat3 DNA binding domain, and Colivelin is a potent synthetic peptide activator of pStat3, which increases pStat3 levels by acting through the GP130/IL6ST complex”, and the rationale has been stated in lines 32--322 as “To functionally demonstrate that pStat3 signaling is downstream of Trpv4, rescue experiments were performed by injecting into ovaries with individual and combined small molecules”.

(7) Discussion of evolutionary differences: The Discussion misses an important opportunity to address why Stat3 activates kdm6b in ricefield eel but represses it in turtles. It is difficult to reconcile how the same transcription factor could exert opposite effects on the same gene during sex determination without additional context. A comparison of kdm6b regulation and sequence conservation between turtles and ricefield eel would strengthen this section.

We have downloaded the promoter sequences of red eared turtle and ricefield eel. Based on the DNA sequences (Author response image 3), the similarity (conservation) was low between the two species.

**Author response image 3.** 

It was appeared that DNA around the Stat3 binding sites in turtle are GC rich (CpG island), which may be subjected to DNA methylation modification, whereas the DNA in ricefield eel are not GC rich.The observations imply that the role of pStat3 is to promote the repression of kdm6b in turtle but the activation of kdm6b in ricefield eel.

Moreover, our unpublished data showed that Trpv4-controlled calcium signaling is required to remove the repressive histone modification H3K27me3 at the *kdm6b* gene. If pStat3 is downstream of Trpv4 in this case, it supports again that Trpv4-pStat3 axis activate kdm6b in ricefield eel.

Warm temperature promotes female sex in turtle but male sex in ricefield eel. If pStat3 is mediating Trpv4, it is not surprising that it represses kdm6b in turtle but activate it in ricefield eel.

Based on above, we have added some sentences in the discussion part, and it reads “We reasoned that a yet-unidentified co-factor may determine whether Stat3 is a transcriptional repressor or activator. A comparison of promoter sequences of kdm6b between turtle and ricefield eel supported this”.

(8) Supplementary figure formatting: Supplementary figures should be provided in accordance with eLife formatting guidelines.

We have now formatted the supplementary figures that are in accordance with eLife formatting requirement. Please see the new uploaded supplementary figures.

In sum, the interpretations are still limited by the above concerns regarding data presentation and reagent specificity.

We thank our editor for the inspiring comments. We believe we have addressed all the major concerns by our editor.
